# The role of automaticity and attention in neural processes underlying empathy for happiness, sadness, and anxiety

**DOI:** 10.3389/fnhum.2013.00160

**Published:** 2013-05-08

**Authors:** Sylvia A. Morelli, Matthew D. Lieberman

**Affiliations:** ^1^Department of Psychology, Stanford UniversityStanford, CA, USA; ^2^Department of Psychology, University of California Los AngelesLos Angeles, CA, USA

**Keywords:** empathy, attention, automaticity, cognitive load, fMRI, happiness, sadness, anxiety

## Abstract

Although many studies have examined the neural basis of empathy, relatively little is known about how empathic processes are affected by different attentional conditions. Thus, we examined whether instructions to empathize might amplify responses in empathy-related regions and whether cognitive load would diminish the involvement of these regions. Thirty-two participants completed a functional magnetic resonance imaging session assessing empathic responses to individuals experiencing happy, sad, and anxious events. Stimuli were presented under three conditions: watching naturally, actively empathizing, and under cognitive load. Across analyses, we found evidence for a core set of neural regions that support empathic processes (dorsomedial prefrontal cortex, DMPFC; medial prefrontal cortex, MPFC; temporoparietal junction, TPJ; amygdala; ventral anterior insula, AI; and septal area, SA). Two key regions—the ventral AI and SA—were consistently active across all attentional conditions, suggesting that they are automatically engaged during empathy. In addition, watching vs. empathizing with targets was not markedly different and instead led to similar subjective and neural responses to others' emotional experiences. In contrast, cognitive load reduced the subjective experience of empathy and diminished neural responses in several regions related to empathy and social cognition (DMPFC, MPFC, TPJ, and amygdala). The results reveal how attention impacts empathic processes and provides insight into how empathy may unfold in everyday interactions.

## Introduction

Empathy allows us to understand and share others' emotions, creating a bridge between the self and the innermost experiences of another person. As we interact with others in our everyday lives, we may respond empathically to one person, but fail to connect with how another person is feeling. While previous research has suggested that certain factors—such as similarity to the target and familiarity with an experience—can trigger empathy (Preston and De Waal, 2002; Mitchell et al., 2006; Xu et al., 2009), very little research has examined how attention impacts our ability to empathize. Past research suggests that empathy may occur instantaneously and automatically when we recognize another's emotional state (Preston and De Waal, 2002), even if we are cognitively busy. However, other research suggests that empathy is disrupted when we are distracted and cognitively occupied (Gu and Han, 2007). Because attentional resources are often depleted during everyday interactions, it is important to know if empathy is automatically engaged or requires controlled and effortful processing. Thus, the current study examines the role of automaticity and attention in neural processes underlying empathy.

### Core neural regions for empathy

A key reason to look at empathy for multiple emotions under a variety of attentional conditions is that it allows for an analysis of core neural regions for empathy. Previous research has identified neural regions that are consistently activated during empathy for physical pain (i.e., dorsal anterior cingulate cortex, dACC; and anterior insula, AI) (Morrison et al., 2004; Singer et al., 2004; Botvinick et al., 2005; Jackson et al., 2005; Zaki et al., 2007; Xu et al., 2009; Lamm et al., 2011). These reliable activations in the dACC and AI have led some researchers to conclude that these regions are part of a core network in empathy (Fan et al., 2011). However, it is unknown whether the dACC and AI are essential to empathic processes more generally (i.e., not just empathy for pain) and whether these regions are activated during empathy for both positive and negative emotions.

Recent neuroimaging research suggests that other neural regions—such as the medial prefrontal cortex (MPFC; BA 10), dorsomedial prefrontal cortex (DMPFC; BA 9), and ventromedial prefrontal cortex (VMPFC; BA 11)—may be involved in empathic processes. For example, accurate empathic judgments are associated with increased MPFC activity (Zaki et al., 2009). MPFC is also consistently activated in mentalizing or theory of mind tasks in which participants infer the mental states of others (Frith and Frith, 2006). In addition, empathy for social and emotional pain activates both MPFC and DMPFC (Masten et al., 2011; Bruneau et al., 2012; Meyer et al., 2012). For patients with neurodegenerative disease, atrophy in MPFC and DMPFC is associated with empathic deficits (Rankin et al., 2003, 2006). In addition, lesion patients with profound empathy deficits have damage in VMPFC (Shamay-Tsoory et al., 2003). Perspective-taking, a key component of empathy, also activates DMPFC (D'Argembeau et al., 2007) and VMPFC (Ames et al., 2008). Finally, judging the emotional states of others increases MPFC, DMPFC, and VMPFC activity (Farrow et al., 2001). Notably, many of these studies did not examine empathy for physical pain and instead focused on neural responses during empathy for other emotions (e.g., social pain). Thus, MPFC, DMPFC, and VMPFC may be involved in empathic processing more generally and may not have been implicated in previous research due to an exclusive focus on empathy for pain.

Additionally, we posit that empathy may increase prosocial motivation and neural activity in SA. In fact, numerous animal studies have demonstrated that the septal area is critical for maternal caregiving (Stack et al., 2002; Gammie, 2005). Recent analyses on a subset of this data also provide tentative evidence that SA activation during empathy predicts daily prosocial behavior in humans (Morelli et al., in press). In addition, past fMRI research has shown that SA activity is related to prosocial behavior, such as charitable donations and providing support to others (Krueger et al., 2007; Inagaki and Eisenberger, 2012; Moll et al., 2011; Eisenberger and Cole, 2012). Thus, we speculate that the septal area, along with DMPFC, MPFC, and VMPFC, may be a core neural region for empathy. The current study examined these and other regions during empathy for three emotions (happiness, sadness, and anxiety), in order to identify regions commonly active during empathy.

### Empathy under different attentional conditions

Relatively little is known about the operational characteristics of empathy and how empathic processes are affected by different attentional conditions. Does being under cognitive load alter the degree of empathy a person feels? The influential Perception-Action Model of empathy suggests that empathy should not be affected by cognitive load (Preston and De Waal, 2002). Preston and De Waal (2002) wrote “attended perception of the object's state automatically activates the subject's representations of the state, situation, and object, and that activation of these representations automatically primes or generates the associated autonomic and somatic responses, unless inhibited” (p. 4). By this account, seeing someone else in an emotional state *automatically* generates emotion in the perceiver, regardless of cognitive load. Perhaps influenced by this statement, very few fMRI studies of empathy have asked participants to do anything besides passively watch empathically-relevant video or images.

Three studies have looked at cognitive load effects, all showing reduced neural responses in empathy-related regions (i.e., dACC, AI, MPFC) (Gu and Han, 2007; Fan and Han, 2008; Rameson et al., 2012). However, Rameson et al. (2012) also observed that those individuals highest in trait empathy showed no reductions, neurally or experientially, under load. In addition, Fan and Han (2008) demonstrated that an early component of empathic neural responses is unaffected by cognitive load, whereas a later component of empathic neural responses is dampened by cognitive load. Thus, the present study aims to more thoroughly explore this question and to examine how cognitive load impacts empathy for a variety of emotional experiences (i.e., happiness, sadness, and anxiety). Based on past research, we hypothesized that regions related to controlled processes, such as mentalizing (e.g., MPFC), would be reduced under cognitive load (Rameson et al., 2012). In addition, we posited that cognitive load would dampen affective responses to the targets, reducing activity in regions associated with positive affect during empathy for happiness (e.g., VMPFC) and regions associated with negative affect during empathy for sadness and anxiety (e.g., dACC and AI) (Morelli et al., in press).

While cognitive load instructions might diminish empathy-related processes that are not fully automatic, other instructions might amplify responses in those same regions. Although some studies have explicitly focused participants' attention on the experience of a target individual or the similarity between the observer and target (Lamm et al., 2007; Sheng and Han, 2012), studies have not typically compared neural responses during directed empathy instructions relative to passive watching instructions. Such a comparison is important not only because it can highlight the attentional malleability of empathic processes, but also because it can help characterize what participants are actually doing when unconstrained during passive watching. We previously reported on this comparison in the context of empathy for sadness and found no differences in dACC and insula, but found significantly greater MPFC activity during instructed empathizing compared to passive watching (Rameson et al., 2012). In the current study, we expand on this analysis to include a comparison of passive watching and instructed empathizing with three emotions (happiness, sadness, and anxiety). Based on past research, we predicted that instructions to empathize would amplify neural responses in regions related to mentalizing (e.g., MPFC), as well as affect-related regions (e.g., dACC, AI, and VMPFC).

### Overview

In our past work, parts of the present dataset have been analyzed, and the results have begun to address some of these outstanding questions. For example, we have previously examined how cognitive load affects neural and behavioral responses during empathy for sadness (Rameson et al., 2012). In addition, we compared neural responses when participants were instructed to empathize versus passively observe others' sadness (Rameson et al., 2012). More recently, we also examined neural similarities and differences when participants actively empathized with positive emotions (i.e., happiness) and negative emotions (i.e., pain and anxiety) (Morelli et al., in press). However, we have not comprehensively assessed how different attentional conditions may impact neural and behavioral responses during empathy for happiness, sadness, and anxiety. Further, none of the current analyses have been previously published and represent a novel and systematic approach to addressing our key questions.

More specifically, the main goal of the current study was to explore how neural activity during empathy is affected by different attentional conditions (i.e., watching, empathizing, and under cognitive load). By measuring neural activity during empathy for various emotions, we first aimed to pinpoint core neural regions that are activated whenever one might be experiencing empathy. We then examined whether observing others' emotional experiences (i.e., watch instructions) engaged similar or different neural regions than actively empathizing with others' emotional experiences (i.e., empathize instructions). We also tested if cognitive load would diminish the involvement of core neural regions for empathy. Lastly, we examined what neural regions were automatically engaged during empathy and active across all attentional conditions.

## Methods

### Participants

Informed consent was obtained from 32 healthy, right-handed undergraduates (16 male; mean age = 19.9, *SD* = 1.4) who were told the purpose of the study was to learn how emotion is processed in the brain. A subset of the data from these same participants has been previously reported (Morelli et al., in press; Rameson et al., 2012).

### Procedure

Participants completed a functional magnetic resonance imaging (fMRI) empathy task using naturalistic stimuli, specifically photos of individuals in happy, sad, anxious, and neutral situations. Stimuli were presented under three conditions: watching naturally (*watch*), actively empathizing (*empathize*), and under cognitive load (*memorize*; memorizing an 8-digit number). After exiting the MRI scanner, participants rated their empathic concern for targets in the empathy task.

### Empathy task in MRI scanner

#### Conditions

**Figure 1:**
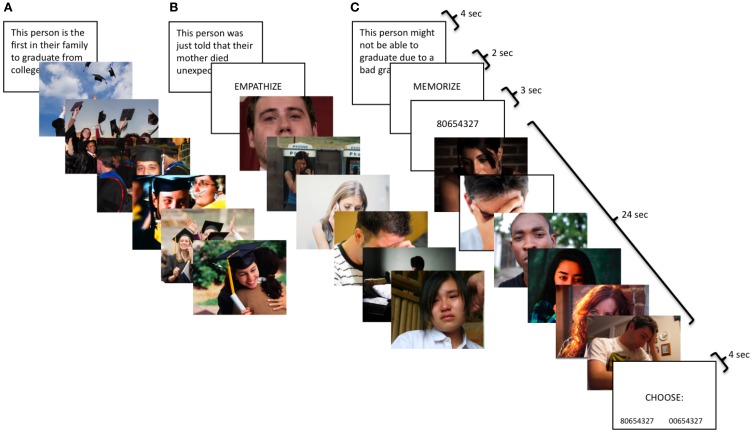
**Participants viewed naturalistic stimuli with three types of instructions: (A) watch, (B) empathize, and (C) memorize combined with three different emotions: (1) happiness, (2) sadness, and (3) anxiety.** Therefore, participants saw nine different block types: happy watch, sad watch, anxiety watch, happy empathize, sad empathize, anxiety empathize, happy memorize, sad memorize, and anxiety memorize.

In the neutral condition, participants viewed blocks of photos with people performing everyday non-emotional actions (e.g., ironing, cutting vegetables). For all other conditions, participants completed an empathy task involving three emotions—happiness, sadness, and anxiety—and three types of instructions—watch, empathize, and memorize. Each block consisted of a contextual sentence describing a situation followed by six photos depicting different individuals in that situation (Figure 1). Happy situations included events like being hired for one's dream job or being the first person in the family to graduate from college. Examples of sad situations were attending a loved one's funeral or being fired from a job. Anxiety situations described events such as potentially not graduating due to a bad grade or being medically examined for a serious illness.

#### Photo stimuli

For the neutral condition, the photo stimuli were adapted from Jackson et al. (2005). For all other conditions, the photo sets were developed by the authors. Within each block, half of the targets were male and half female. An arrow indicated the target individual if a photo depicted several people. Images were equated across conditions on arousal, valence, luminance, and complexity, and sentences were equated on length. Images were selected from a larger pool in order to equate them on a number of features. Blocks were equated across instruction type on arousal, luminance, complexity, and the number of letters in each contextual sentence preceding that block. Subjective ratings of valence and arousal were made by 16 (8 male) undergraduate pilot judges. Raters judged the valence of each photo on a scale from 1 (*very negative*) to 7 (*very positive*), and arousal on a scale from 1 (*very weak*) to 7 (*very strong*). Luminance was measured using Adobe Photoshop CS. Complexity was determined using the size of each image in jpeg (compressed) format (Calvo and Lang, 2004). In previous research, compressed image file sizes have been shown to be highly correlated with both subjective measures of complexity (Donderi, 2006; Tuch et al., 2009) and objective visual search performance (Donderi and McFadden, 2005).

#### Task instructions

For all conditions, participants were told photos depicted real events drawn from news stories, documentaries, and blogs. For the neutral condition, participants were simply asked to look at the photos for the whole time they were on the screen. For the watch condition, participants were instructed to respond to the photos naturally, as if they were at home and had come across the images in a magazine. For the empathize condition, participants were told to take each target's perspective and imagine how he/she felt about the situation and how it affected his/her life. These instructions have previously been shown to induce empathic concern (Toi and Batson, 1982). For the memorize condition, participants were told to keep an 8-digit number in memory while looking at the images.

#### Task timing and display order

The neutral condition consisted of four blocks; each block displayed 16 neutral photos for 2 s each. For the empathy task, each emotion had a total of nine blocks, divided into three instruction types: watch (3 blocks), empathize (3 blocks), and memorize (3 blocks). For the watch blocks, the contextual sentence was displayed for 4 s, followed by 6 photos presented for 4 s each. The empathize blocks displayed the contextual sentence for 4 s, followed by the instruction to “empathize” for 2 s, then ended with 6 photos for 4 s each. For memorize blocks, the contextual sentence was displayed for 4 s, followed by the cue to “memorize” for 2 s, then an 8-digit number for 3 s, then the block of 6 photos for 4 s each, and finally a memory test for the number for 4 s. Participants chose between the correct number and a number that was identical except for one digit. For all conditions, each block was separated by a 12-s rest period. The first run consisted exclusively of three watch blocks for each emotion, as this instruction type was meant to capture unprimed, spontaneous reactions. In the next two runs, participants were cued to trial type by the word “empathize” or “memorize,” which appeared for 2 s after each sentence. Three empathize blocks and three memorize blocks were included for each emotion, intermixing empathize and memorize blocks across the two runs. Lastly, the third run included the four neutral blocks.

### fMRI acquisition and data analysis

Scanning was performed on a Siemens Trio 3T. Functional images were acquired using an EPI gradient-echo sequence (*TR* = 2000 ms, *TE* = 30 ms, 4 mm slice thickness/no gap, *FOV* = 19.2 cm, matrix = 64 × 64, flip angle = 90°). A T2-weighted structural image was acquired coplanar with the functional images (*TR* = 5000 ms, *TE* = 34 ms, 4 mm slice thickness/no gap, *FOV* = 19.2 cm, matrix = 128 × 128, flip angle = 90°). All images were scalped using the Brain Extraction Tool of FSL (FMRIB Software Library; Oxford University, Oxford, UK) and realigned within runs using MCFLIRT. Images were then checked for residual motion and noise spikes using a custom automated diagnostic tool (thresholded at 2 mm motion or 2% global signal change from one image to the next). In SPM8 (Wellcome Department of Imaging Neuroscience, London), all functional and anatomical images were reoriented to set the origin to the anterior commissure and the horizontal (*y*) axis parallel to the AC-PC line. Also in SPM 8, functional images were realigned within and between runs to correct for residual head motion, and coregistered to the matched-bandwidth structural scan using a 6-parameter rigid body transformation. The coregistered structural scan was then normalized into Montreal Neurological Institute (MNI) standard stereotactic space using the scalped ICBM152 template and the resulting parameters were applied to all functional images. Finally, the normalized functional images were resliced into voxels of 3 mm^3^ and smoothed using an 8 mm full width at half maximum Gaussian kernel.

All single subject and group analyses were performed in SPM8. First-level effects were estimated using the general linear model and employing a canonical hemodynamic response function convolved with the experimental design. Low-frequency noise was removed using a high-pass filter. Group analyses were conducted using random-effects models to enable population inferences (Nichols et al., 2005). To keep all instruction types as well-constrained and equivalent as possible, empathize, watch, and memorize trials were modeled using only the 24 s of image presentation that was invariant across instruction types. The remaining trial elements—the instruction prompts, contextual sentences, 8-digit number presentation and memory test (for memorize blocks)- were modeled separately and were not included in the baseline condition. In addition, the neutral condition was modeled using only the 32 s of image presentation for each neutral block.

#### Whole-brain group-level analyses

Whole-brain group-level analyses were performed using an uncorrected *p*-value of <0.005 with a cluster threshold of 43 based on a Monte Carlo simulation in AFNI's Alphasim effectively producing an FDR of *p* = 0.05 (Lieberman and Cunningham, 2009). For visualization of results, group contrasts were overlaid on a surface representation of the MNI canonical brain using the SPM surfrend toolbox and NeuroLens (http://spmsurfrend.sourceforge.net; http://www.neurolens.org/NeuroLens/Home.html).

#### Masked regions of interest analyses

Masked regions of interest (ROI) analyses were conducted using SPM8. Anatomical ROIs were created for regions commonly involved in empathy (dACC and AI), emotion (SA; amygdala; and rostral anterior cingulate cortex, rACC), and mentalizing (DMPFC, MPFC, and TPJ). Anatomical ROIs were constructed using the Wake Forest University Pickatlas Tool (Maldjian et al., 2003) with the Automated Anatomical Labeling Atlas (AAL; Tzourio-Mazoyer et al., 2002) or using Marsbar (http://marsbar.sourceforge.net).

A cingulate ROI that combined Brodmann Areas (BA) 24 and 32 (dilated to 2 mm) as well as the AAL anterior, middle, and posterior cingulate was divided into the dACC (bounded between *y* = 33 and *y* = 0) and the rACC (bounded between *y* = 54 and *y* = 34) (Bush et al., 2002; Vogt et al., 2003; Beckmann et al., 2009). AAL insula was bounded caudally at *y* = 0 to include only the anterior region and did not include pars opercularis, pars triangularis, or pars orbitalis. The SA ROI consisted of a box that extends from *x* = −6 to *x* = 6, *y* = −2 to *y* = 0, and *z* = 0 to *z* = 10, and is based on the Atlas of the Human Brain (Mai et al., 2004). The amygdala ROI was taken directly from AAL.

The MPFC and DMPFC ROIs were manually constructed in FSLview in a voxel-by-voxel fashion, informed by recent meta-analyses and reviews pertaining to MPFC function (both anterior rostral and dorsal aspects) and using the AAL labeling scheme as implemented in the WFU Pickatlas for comparison and reference (Steele and Lawrie, 2004; Amodio and Frith, 2006; Northoff et al., 2006). The DMPFC ROI was bounded ventrally at *z* = 26 to distinguish from MPFC, laterally at *x* = ±20 to include only the medial aspect, and caudally at *y* = 44 to exclude anterior cingulate. The MPFC ROI was bounded dorsally at *z* = 24 to distinguish from DMPFC, ventrally at *z* = −10 to distinguish from VMPFC, laterally at *x* = ±20 to include only the medial aspect, and caudally at *y* = 46 to exclude anterior cingulate. The TPJ ROI was created using the union of BA 22, 39, and 40, bounded between *x* = ±38, *y* = −40 and −68, and *z* = 22 and 38 (Decety and Lamm, 2007).

An overall mask for all cortical ROIs was submitted to Monte Carlo simulations, which determined that an uncorrected *p*-value of 0.005 with a cluster threshold of 28 voxels yielded a *p* < 0.05 FDR correction. Because subcortical regions tend to be substantially smaller, individual masks were created for SA and amygdala. Monte Carlo simulations indicated that for these smaller regions an uncorrected *p*-value of 0.005 with a cluster threshold of 3 voxels provided the same FDR correction.

### Post-scanner empathy ratings

Immediately post-scan, participants rated their empathic reaction to each block in the empathy task. Participants viewed the original task again, but with shorter presentation times (1 s per image) and without the neutral condition. Participants were told to remember how they felt when they first saw the images. For happy blocks, participants rated how happy they were for the targets on a scale from 1 (*not at all*) to 7 (*very much*). For sad and anxiety blocks, participants rated how concerned they felt for the targets on a scale from 1 (*not at all*) to 7 (*very much*). Participants were told “concerned” meant how compassionate, sympathetic, and moved they felt, as these adjectives have been used to assess empathy in previous research (Toi and Batson, 1982).

## Results

### Post-scanner empathy ratings

**Figure 2:**
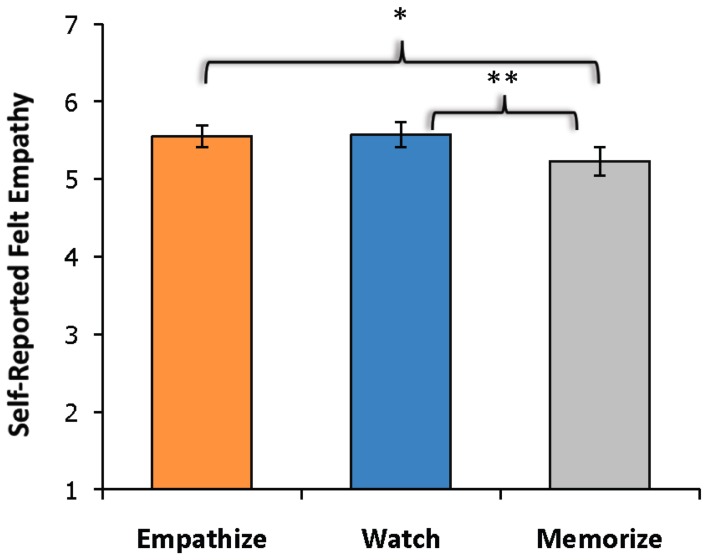
**Self-reported empathy showed a main effect of instruction type with participants reporting less empathy during memorize instructions than during empathize or watch instructions.** The empathize and watch conditions did not differ significantly on self-reported empathy.

Due to technical difficulties, post-scan ratings for three participants were not collected. A three (happy, sad, anxiety) by three (watch, empathize, memorize) repeated-measures ANOVA revealed a main effect of instruction type on experienced empathy, *F*_(2, 56)_ = 29.64, *p* < 0.001, as well as a main effect of emotion type on experienced empathy, *F*_(2, 56)_ = 7.25, *p* < 0.005. However, the interaction between emotion type and instruction type was not significant. Follow-up paired samples *t*-tests showed that participants reported less empathy during memorize blocks (*M* = 5.23, *SD* = 0.96) than during the empathize blocks (*M* = 5.55, *SD* = 0.76), *t*_(28)_ = −2.78, *p* < 0.05, or during the watch blocks (*M* = 5.57, *SD* = 0.84), *t*_(28)_ = −3.30, *p* < 0.005 (Figure 2). Empathize and watch blocks did not differ significantly on reported empathy. Participants also reported experiencing reduced empathy for anxiety (*M* = 4.97, *SD* = 0.90) compared to happiness (*M* = 5.67, *SD* = 0.84), *t*_(28)_ = −5.67, *p* < 0.001, and to sadness (*M* = 5.70, *SD* = 0.87), *t*_(28)_ = −9.00, *p* < 0.001. Self-reported empathy did not differ significantly for happiness and sadness.

### fMRI results

#### Behavioral performance during memorize blocks

Accuracy rate was 84% (*SD* = 20%) for the memory test after each memorize block, indicating that participants were performing the memory task as intended.

#### Overview of effects

Given that our 3 × 3 experimental design yielded many potential comparisons, we wanted to provide an overview of the data and identify patterns across the nine cells of our design. Therefore, we looked for effects in the eight ROIs for each of the nine conditions compared to the neutral condition. We conducted masked ROI analyses using regions commonly involved in empathy (dACC and AI), emotion (SA, amygdala, and rACC), and mentalizing (DMPFC, MPFC, and TPJ).

Table 1 shows a summary of regions that produced significant activations for each of the nine cells of our design and reveals a number of interesting patterns. Regions related to mentalizing (DMPFC, MPFC, and TPJ) produced reliable activations during empathize and watch instructions, but were not activated during memorize instructions. Somewhat surprisingly, the amygdala showed the same pattern. In contrast, dACC was reliably present during memorize instructions, but only appeared in two of the six remaining non-memorize blocks. Finally, SA activations were present during all nine trial types, and AI activations were present during eight of the nine trial types. Out of the 8 ROIs, the only the SA and AI were consistently activated across conditions. rACC was also observed in five of the nine trial types, but with no particular pattern with respect to emotion or attentional instructions.

**Table 1:** **Patterns of neural activity for each instruction type (compared to viewing neutral photos) within anatomically-defined regions of interest previously associated with empathy, emotion, and mentalizing**.

	**dACC**	**AI**	**Septal**	**Amygdala**	**rACC**	**DMPFC**	**MPFC**	**R TPJ**
**EMPATHIZE**								
Happy			•	•		•	•	•
Sad		•	•	•	•	•	•	•
Anxiety	•	•	•	•	•		•	
**WATCH**								
Happy		•	•	•	•	•	•	•
Sad	•	•	•	•		•	•	•
Anxiety		•	•	•		•	•	•
**MEMORIZE**								
Happy	•	•	•		•			
Sad	•	•	•					
Anxiety	•	•	•		•			

Note. Cells were marked using a threshold of p < 0.005 and a 28 voxel extent which provides FDR corrected p < 0.05. Separate ROI masks were created for the septal area and amygdala. In these regions, marked cells are significant at p < 0.005 and a 3 voxel extent (p < 0.05 FDR corrected). For anterior insula and amygdala, cell are marked if a significant cluster appeared in either hemisphere.

#### Common activations during empathy for happiness, sadness, and anxiety

Our first goal was to identify core neural regions that were activated across different kinds of empathic experiences. To determine whether any neural regions were commonly recruited when trying to empathize with each of three different emotions, we used a conjunction analysis (Nichols et al., 2005) for the comparison of the empathize condition to the neutral condition for each of the three emotion types (happiness, sadness, and anxiety). This method only yielded clusters that were significantly active in each of the three contributing contrasts.

**Figure 3:**
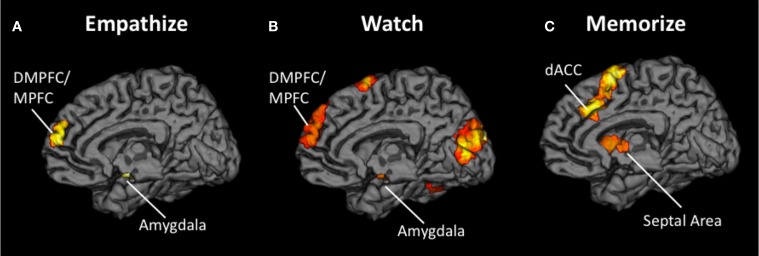
**Neural overlap during empathy for happiness, sadness, and anxiety using conjunction analyses for the contrasts (A) Happy Empathize > Neutral, Sad Empathize > Neutral, and Anxiety Empathize > Neutral (B) Happy Watch > Neutral, Sad Watch > Neutral, and Anxiety Watch > Neutral, and (C) Happy Memorize > Neutral, Sad Memorize > Neutral, and Anxiety Memorize > Neutral.** In both the empathize and watch conjunction analyses, DMPFC and MPFC were two of the common neural areas across emotions. However, DMPFC and MPFC did not appear in the memorize conjunction analysis; instead, dACC and AI were two of the common neural areas across emotions.

First, a contrast image was created for each emotion type that compared empathize instructions to the neutral condition (i.e., Happy Empathize > Neutral, Sad Empathize > Neutral, and Anxiety Empathize > Neutral). Then, a conjunction analysis of all three contrast images was used to identify neural regions that were commonly recruited when empathizing with the three emotions. This conjunction analysis across emotion types revealed common activity in MPFC, DMPFC, and amygdala, regions typically associated with mentalizing and emotion (see Figure 3A, Table 2). Slightly lowering the voxel extent for this contrast also revealed activation in SA (with the peak voxel at *x* = 3, *y* = 2, *z* = 4; *t* = 3.51; *k* = 38).

**Table 2:** **Neural regions that were commonly activated during happiness, sadness, and anxiety for empathize compared to neutral, watch compared to neutral, and memorize compared to neutral**.

**Region**	**BA**	**Hemisphere**	***K***	**Coordinates**			***t***
				***x***	***y***	***z***	
**CONJUNCTION OF HAPPY EMPATHIZE > NEUTRAL, SAD EMPATHIZE > NEUTRAL, AND ANXIETY EMPATHIZE > NEUTRAL**							
Medial prefrontal cortex/dorsomedial prefrontal cortex	10/9	R	70	6	59	13	3.86
Amygdala	–	R	61	18	−4	−11	4.41
		L	46	−21	−7	−11	5.01
**CONJUNCTION OF HAPPY WATCH > NEUTRAL, SAD WATCH > NEUTRAL, AND ANXIETY WATCH > NEUTRAL**							
Medial prefrontal cortex	10	R	421^1^	6	59	16	3.70
Dorsomedial prefrontal cortex	9/8	R	421^1^	3	56	31	3.93
Temporoparietal junction/posterior superior temporal sulcus	40	R	101	54	−43	16	4.30
Anterior insula	13	L	64^2^	−42	14	−17	3.79
Amygdala	–	R	49	18	−7	−14	4.33
Ventrolateral prefrontal cortex	47	L	64^2^	−45	29	−2	3.97
Dorsal premotor cortex	6	R	64	6	11	67	5.15
Fusiform	37	R	44	42	−55	−14	5.09
Occipital lobe	19/18	–	387	−6	−97	25	6.97
**CONJUNCTION OF HAPPY MEMORIZE > NEUTRAL, SAD MEMORIZE > NEUTRAL, AND ANXIETY MEMORIZE > NEUTRAL**							
Septal area	–	L	55	−3	−4	1	3.41
Dorsal anterior cingulate cortex	32	R	500^3^	3	29	31	4.42
Anterior insula	13	R	199	39	23	10	5.28
		L	223	−33	23	4	6.36
Dorsal premotor cortex	6	L	500^3^	−6	2	64	5.87

Note. BA refers to putative Brodmann's Area; L and R refer to left and right hemispheres; k refers to the cluster size (in voxels); x, y, and z refer to MNI coordinates in the left-right, anterior-posterior, and inferior-superior dimensions, respectively; t refers to the t-score at those coordinates (local maxima). Regions with ks that share a superscript originate from the same cluster.

Similarly, the conjunction analysis across emotion types when watching others' emotional experiences (i.e., a conjunction of Happy Watch > Neutral, Sad Watch > Neutral, and Anxiety Watch > Neutral) produced common activations in a variety of regions related to social cognition (i.e., MPFC, DMPFC, TPJ, and pSTS), as well as in ventral AI and amygdala (see Figure 3B, Table 2). Lowering the voxel extent for this contrast once again revealed activation in SA (with the peak voxel at *x* = 0, *y* = −4, *z* = −2; *t* = 3.31; *k* = 16).

In contrast, when participants viewed the same kinds of emotional scenes but were focused on memorizing an 8-digit number, mentalizing-related regions were not commonly activated across emotion types. Instead, the conjunction of Happy Memorize > Neutral, Sad Memorize > Neutral, and Anxiety Memorize > Neutral yielded common activity in SA and in regions associated with controlled processes and salience detection: dACC and dorsal AI (see Figure 3C, Table 2). Taken together, these results suggest that regions related to mentalizing and emotion may be critical for generating empathic responses. However, cognitive load may disrupt activity in these core regions and reduce empathic responding.

#### Neural similarities and differences between empathizing and watching

**Figure 4:**
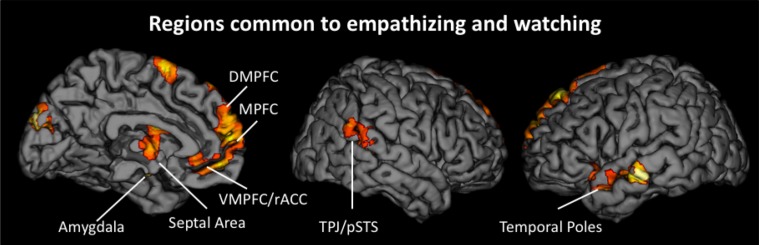
**Neural regions that were commonly activated during the empathize and watch conditions (collapsing across emotions) compared to neutral**.

To determine if reacting naturally (i.e., watching) and trying to empathize activated common neural regions, we ran additional conjunction analyses. For these analyses, we collapsed all empathize blocks into one condition and all watch blocks into one condition, regardless of emotion. We then created a contrast image that compared empathize instructions to the neutral condition (i.e., Empathize > Neutral) and another contrast that compared watch instructions to the neutral baseline (i.e., Watch > Neutral). A conjunction analysis of these two contrast images was then used to identify neural regions that were commonly recruited when trying to empathize or simply watch. This conjunction analysis showed activity in regions previously associated with social cognition, including the MPFC, DMPFC, VMPFC/rACC, TPJ, pSTS, and temporal poles, in addition to regions related to emotion, including SA, amygdala, and ventral AI (Table 3 and Figure 4).

**Table 3:** **Neural regions that were commonly activated during empathize and watch (collapsed across happiness, sadness, and anxiety) compared to neutral**.

**Region**	**BA**	**Hemisphere**	***k***	**Coordinates**			***t***
				***x***	***y***	***z***	
**CONJUNCTION OF EMPATHIZE > NEUTRAL AND WATCH > NEUTRAL**							
Medial prefrontal cortex	10	R	713^1^	6	62	13	5.21
Dorsomedial prefrontal cortex	8/9	R	713^1^	6	56	28	4.30
Ventromedial prefrontal cortex/rostral anterior cingulate cortex	11/32	L	713^1^	−3	47	−11	4.51
Temporoparietal junction/posterior superior temporal sulcus	40	R	148	51	−40	10	5.20
Temporal poles/middle temporal gyrus	38/21	L	275^2^	−54	2	−17	3.96
Septal area	–	R	150	3	−1	−2	4.03
Anterior insula	13	L	275^2^	−42	14	−17	5.50
Amygdala/hippocampus	–	R	119	18	−7	−11	5.54
		L	275^2^	−18	−10	−14	6.21
Dorsal premotor cortex	6	R	112	3	8	67	5.91
Occipital lobe	18/19	L	151	−3	−91	28	5.35

Note. BA refers to putative Brodmann's Area; L and R refer to left and right hemispheres; k refers to the cluster size (in voxels); x, y, and z refer to MNI coordinates in the left-right, anterior-posterior, and inferior-superior dimensions, respectively; t refers to the t-score at those coordinates (local maxima). Regions with ks that share a superscript originate from the same cluster.

To identify differences between empathize instructions and watch instructions, we compared the empathize and watch conditions (Table 4). We did not find a large number of neural differences between the two instruction types, which is consistent with our finding that self-reported empathy was at similar levels for each instruction type. For the contrast Watch > Empathize, there was increased activation in some regions related to social cognition, namely DMPFC, precuneus, and pSTS. However, it appears that trying to empathize and watching naturally may have more neural similarities than differences.

**Table 4:** **Neural regions that were more active for empathize compared to watch (collapsing across emotions), as well as neural regions that were more active for watch compared to empathize (collapsing across emotions)**.

**Region**	**BA**	**Hemisphere**	***k***	**Coordinates**			***t***
				***x***	***y***	***z***	
**EMPATHIZE > WATCH**							
Dorsal anterior cingulate cortex	32/24	L	289^1^	−9	11	34	4.06
Supplementary motor area	6	R	289^1^	9	−7	55	4.36
Putamen	–	L	59	−18	11	−8	4.43
Precentral gyrus	6	L	48	−21	−16	76	3.97
Postcentral gyrus	1/2	R	89	57	−22	55	3.39
**WATCH > EMPATHIZE**							
Dorsomedial prefrontal cortex	8/9	−	229	0	56	40	4.10
Precuneus	7/31	R	2870^2^	6	−67	40	3.93
Posterior superior temporal sulcus/middle temporal gyrus	22	R	100	51	−43	−2	4.61
		L	158	−63	−40	1	4.67
Dorsolateral prefrontal cortex	8/9/10	R	788	45	35	37	6.07
Inferior parietal lobule/superior parietal lobule	40/7/39	R	726	42	−52	49	5.63
		L	706	−45	−52	40	4.95
Fusiform	37	R	2870^2^	45	−55	−17	3.87
		L	2870^2^	−42	−55	−20	3.68
Occipital lobe	18/19	R	2870^2^	6	−79	1	4.28
Cerebellum	–	L	2870^2^	−3	−82	−26	6.85

Note. BA refers to putative Brodmann's Area; L and R refer to left and right hemispheres; k refers to the cluster size (in voxels); x, y, and z refer to MNI coordinates in the left-right, anterior-posterior, and inferior-superior dimensions, respectively; t refers to the t-score at those coordinates (local maxima). Regions with ks that share a superscript originate from the same cluster.

#### Cognitive load effects

**Figure 5:**
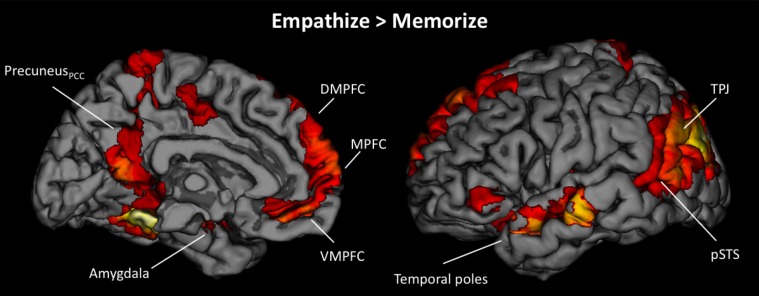
**Neural regions that showed reduced activity under cognitive load compared to empathizing (Empathize > Memorize)**.

Next, we wanted to more directly test whether cognitive load (i.e., memorize blocks) would diminish the involvement of neural regions that were active when empathizing or watching naturally. Because we were primarily interested in the effect of cognitive load, the following analyses collapse all empathize blocks into one condition, all watch blocks into a second condition, and all memorize blocks into a third condition. To identify what regions were less active under load compared to actively empathizing, we compared empathize blocks (all emotion types) to memorize blocks (all emotion types) (see Table 5). For this contrast Empathize > Memorize, we found activations in regions typically associated with social cognition (i.e., MPFC, DMPFC, VMPFC, precuneus/posterior cingulate cortex, TPJ, pSTS, and temporal poles) and emotional arousal (i.e., amygdala) (see Figure 5). For the contrast Watch > Memorize, we observed activations in the same set of neural regions (see Table 5).

**Table 5:** **Neural regions that were less active under cognitive load compared to empathize (collapsed across emotions) and less active under cognitive load compared to watch (collapsed across emotions)**.

**Region**	**BA**	**Hemisphere**	***k***	**Coordinates**			***t***
				***x***	***y***	***z***	
**EMPATHIZE > MEMORIZE**							
Medial prefrontal cortex	10	L	1197^1^	−6	62	1	4.03
Dorsomedial prefrontal cortex	8/9	R	1197^1^	3	56	28	5.87
Ventromedial prefrontal cortex	11	–	1197^1^	0	38	−14	6.40
Precuneus/posterior cingulate cortex	31	L	6903^2^	−6	−55	16	5.77
Temporoparietal junction/posterior superior temporal sulcus	22/39	R	6903^2^	57	−49	10	6.51
		L	6903^2^	−42	−70	22	6.53
Temporal pole/middle temporal gyrus	21/38	R	6903^2^	54	−1	−17	8.27
		L	6903^2^	−45	14	−23	6.23
Amygdala	–	R	6903^2^	21	−4	−17	6.61
		L	6903^2^	−21	−7	−17	5.53
Ventrolateral prefrontal cortex	46	R	45	54	38	10	5.37
Supplementary motor area	6	R	750^3^	3	−16	58	3.99
Inferior parietal lobule	40	R	113	57	−28	37	4.65
Hippocampus	–	R	6903^2^	30	−16	−14	6.60
		L	6903^2^	−30	−16	−14	6.21
Fusiform	37	R	6903^2^	24	−40	−14	9.99
		L	6903^2^	−24	−46	−11	10.84
Precentral/postcentral gyrus	6/4	R	750^3^	18	−43	70	4.78
Cerebellum	–	R	127	30	−79	−32	5.84
		L	137	−21	−79	−32	6.52
		L	212	−6	−52	−41	4.50
Occipital lobe	19	R	6903^2^	42	−79	25	12.50
		L	6903^2^	−42	−70	22	6.53
**WATCH > MEMORIZE**							
Medial prefrontal cortex	10	R	1728^4^	3	68	10	5.59
Dorsomedial prefrontal cortex	8/9	R	1728^4^	3	56	40	5.99
Ventromedial prefrontal cortex	11	L	1728^4^	−6	38	−14	5.52
Precuneus	7	R	171	9	−64	70	3.81
Temporoparietal junction/posterior superior temporal sulcus	22/39/40	R	9362^5^	57	−49	10	6.52
		L	9362^5^	−48	−70	19	6.54
Temporal poles	38	L	9362^5^	−54	2	−20	6.05
Amygdala	–	R	9362^5^	30	−10	−14	7.72
Ventrolateral prefrontal cortex	45/46/47	R	1728^4^	57	23	28	5.04
		L	9362^5^	−48	41	−8	6.51
Dorsal premotor cortex	6	L	1728^4^	−9	32	55	5.93
Hippocampus	–	R	9362^5^	27	−16	−11	7.87
Fusiform	37	R	9362^5^	36	−46	−8	7.10
		L	9362^5^	−30	−40	−14	7.89
Middle temporal gyrus	21/22	R	9362^5^	60	−7	−14	7.75
		L	9362^5^	−57	−16	−14	7.43
Angular gyrus	39	R	9362^5^	42	−70	25	8.01
		L	9362^5^	−48	−70	31	8.73
Occipital lobe	19	R	9362^5^	36	−70	7	6.68
		L	9362^5^	−33	−85	31	7.24
Cerebellum	–	L	9362^5^	−24	−79	−32	7.84

Note. BA refers to putative Brodmann's Area; L and R refer to left and right hemispheres; k refers to the cluster size (in voxels); x, y, and z refer to MNI coordinates in the left-right, anterior-posterior, and inferior-superior dimensions, respectively; t refers to the t-score at those coordinates (local maxima). Regions with ks that share a superscript originate from the same cluster.

We also identified regions that were more active under load compared to empathizing (Memorize > Empathize) and more active under load compared to watching naturally (Memorize > Watch) (see Table 6). For both of these contrasts, dACC, AI, VLPFC, DLPFC, dorsal premotor cortex, and supplementary motor area were more active under load. In sum, putting people under cognitive load while looking at emotional stimuli may reduce activity in regions associated with social cognition and emotional arousal and increase neural activity in regions associated with attention and effort (Table 7).

**Table 6:** **Neural regions that were more active under cognitive load compared to empathize (collapsed across emotions) and more active under cognitive load compared to watch (collapsed across emotions)**.

**Region**	**BA**	**Hemisphere**	***k***	**Coordinates**			***t***
				***x***	***y***	***z***	
**MEMORIZE > EMPATHIZE**							
Precuneus	7	R	2289^1^	12	−64	40	6.05
		L	2289^1^	−12	−64	52	4.64
Dorsal anterior cingulate cortex	32/24	R	2732^2^	9	29	31	5.35
Anterior insula	13	R	249	36	17	10	6.09
		L	2732^2^	−36	20	1	6.23
Ventrolateral prefrontal cortex	46/47	L	2732^2^	−36	26	28	4.90
Dorsolateral prefrontal cortex	10	R	2732^2^	33	53	22	6.72
		L	2732^2^	−39	50	22	7.41
Inferior parietal lobule	40	R	406	48	−40	49	5.67
		L	2289^1^	−48	−43	52	6.28
Dorsal premotor cortex/supplementary motor area	6	L	2372^2^	−6	2	61	8.13
Precentral gyrus/inferior frontal gyrus	6/9	L	2732^2^	−54	−7	49	6.40
Middle/superior frontal gyrus	6	R	72	21	8	64	4.45
Middle cingulate	23	–	151	0	−22	28	5.29
Occipital lobe	18	L	2289^1^	−9	−76	4	10.62
Cerebellum	–	R	67	27	−67	−20	4.47
**MEMORIZE > WATCH**							
Precuneus	7	R	56	12	−67	40	4.03
		L	118	−9	−73	43	4.83
Temporoparietal junction	40	L	412^3^	−51	−49	28	4.28
Dorsal anterior cingulate cortex	32/24	R	1111^4^	6	26	31	5.97
Anterior insula	13	R	244	36	20	10	6.64
		L	1544^5^	−30	20	4	7.09
Caudate	–	R	1544^5^	12	8	−2	5.77
		L	1544^5^	−6	5	10	3.71
Dorsolateral prefrontal cortex	10/9	R	389	30	41	37	7.71
		L	1544^5^	−36	38	25	5.94
Ventrolateral prefrontal cortex		L	1544^5^	−39	26	28	5.38
Inferior parietal lobule	40	L	412^3^	−48	−40	49	4.92
Dorsal premotor cortex/supplementary motor area	6	L	1111^4^	−6	2	61	11.85
Precentral gyrus/inferior frontal gyrus	6/9	L	1544^5^	−48	−4	43	8.04
Postcentral gyrus	1/2	L	51	−60	−19	25	5.01
Middle cingulate	23	L	90	−3	−22	31	4.71

Note. BA refers to putative Brodmann's Area; L and R refer to left and right hemispheres; k refers to the cluster size (in voxels); x, y, and z refer to MNI coordinates in the left-right, anterior-posterior, and inferior-superior dimensions, respectively; t refers to the t-score at those coordinates (local maxima). Regions with ks that share a superscript originate from the same cluster.

**Table 7:** **A summary of cognitive load effects that illustrates the relative increases and decreases in activation during empathize and watch compared to memorize (collapsed across emotions)**.

	**dACC**	**AI**	**Septal**	**Amygdala**	**rACC**	**DMPFC**	**MPFC**	**R TPJ**
Empathize > Memorize	↓	↓^*^		↑		↑	↑	↑
Watch > Memorize	↓	↓		↑		↑	↑	↑

Note. ↑indicates a relative increase in activation for the ROI during empathize relative to memorize and watch relative to memorize. ↓indicates a relative decrease in activation for the ROI during empathize relative to memorize and watch relative to memorize.

* In addition to the AI cluster that was more active during memorize, a smaller cluster in AI was also more active during empathize compared to memorize.

#### Automaticity

**Figure 6:**
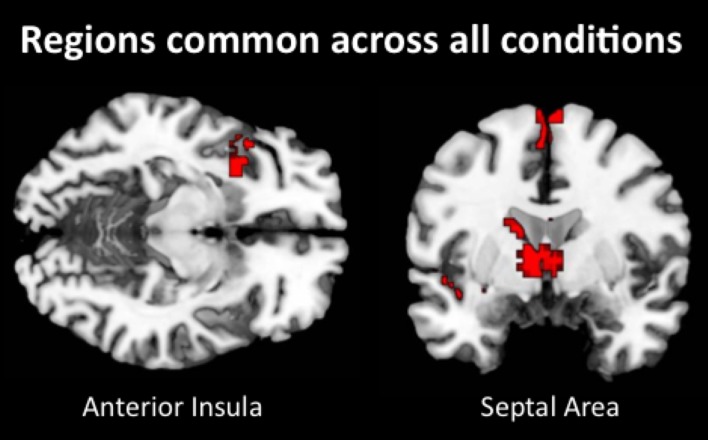
**Neural regions that were commonly activated during empathize, watch, and memorize (collapsed across emotions) relative to neutral**.

Lastly, we examined what neural regions may be automatically engaged during empathy and remain active regardless of the attentional condition. Similar to previous analyses, we collapsed all empathize blocks into one condition, all watch blocks into one condition, and all memorize blocks into one condition. We then created a contrast image that compared empathize instructions to the neutral condition (i.e., Empathize > Neutral), another contrast that compared watch instructions to the neutral condition (i.e., Watch > Neutral), and a final contrast that compared memorize instructions to the neutral condition (i.e., Memorize > Neutral). Finally, a conjunction analysis of these three contrast images was used to identify neural regions that are engaged during all three conditions. This conjunction analysis showed common activity in SA and ventral AI (Table 8 and Figure 6), as well as the dorsal premotor cortex and occipital lobe. Thus, SA and ventral AI seem to be automatically engaged during empathy, regardless of attentional conditions.

**Table 8:** **Neural regions that were commonly activated during empathize, watch, and memorize (collapsed across emotions) compared to neutral**.

**Region**	**BA**	**Hemisphere**	***k***	**Coordinates**			***t***
				***x***	***y***	***z***	
**CONJUNCTION OF EMPATHIZE > NEUTRAL, WATCH > NEUTRAL, AND MEMORIZE > NEUTRAL**							
Septal area	–	R	123	3	−1	−2	4.01
Anterior insula	13	–	53	−39	14	−14	4.64
Dorsal premotor cortex	6	R	91	3	8	67	5.91
Occipital lobe	18/19	L	128	−3	−91	28	5.20

Note. BA refers to putative Brodmann's Area; L and R refer to left and right hemispheres; k refers to the cluster size (in voxels); x, y, and z refer to MNI coordinates in the left-right, anterior-posterior, and inferior-superior dimensions, respectively; t refers to the t-score at those coordinates (local maxima). Regions with ks that share a superscript originate from the same cluster.

## Discussion

The results of the present study begin to address several unanswered questions in the empathy literature. While most studies have examined neural processes during empathy for a single negative emotion, it is unclear whether these neural regions are specific to empathy for each negative emotion or critical for empathic processes more broadly. By measuring empathic processes across multiple emotions, we identified neural regions that are central to an empathic state. We also addressed other gaps in the current research by directly comparing the effects of several attentional conditions (i.e., watch, empathize, memorize) on empathic processes. More specifically, comparing neural responses during empathize and watch instructions allowed us to characterize what participants are actually doing when instructed to observe others (typical instructions in most previous studies). By including cognitive load instructions, we also identified which neural regions are automatically engaged during empathy and which neural regions may be disrupted by reduced attentional resources.

Across analyses, we find evidence for a core set of neural regions that support an empathic state (i.e., DMPFC, MPFC, TPJ, amygdala, AI, and SA). When participants observed or actively empathized with a target, we found relatively consistent activity in regions related to mentalizing (i.e., DMPFC, MPFC, and TPJ) across emotions. Conjunction analyses for each instruction type confirmed this pattern, showing DMPFC and MPFC activation when empathizing and DMPFC, MPFC, and TPJ activation when observing others. While studies on empathy for pain have consistently found dACC and AI activation, our results suggest that regions related to mentalizing may be core neural areas during empathy for both positive and negative emotions.

Previous research demonstrates that DMPFC, MPFC, and TPJ are some of the most consistently activated regions when thinking about the mental states of others (Spreng et al., 2009; Van Overwalle, 2009; Lieberman, 2010). TPJ activation often occurs when reasoning about temporary states such as the goals, intentions, and desires of other people (Saxe and Kanwisher, 2003; Van Overwalle, 2009; Young et al., 2010). Both DMPFC and MPFC are associated with inferring the enduring dispositions of the self and others (Van Overwalle, 2009). Because our task used a variety of emotional and situational contexts, participants probably thought about both the temporary states and enduring dispositions of targets. In addition, the stimuli depicted targets with varied gender, ethnicity, and age, experiencing events that the participants were both familiar and unfamiliar with. Thus, DMPFC may have been activated when participants contemplated targets who were dissimilar to themselves, while MPFC may have been activated when thinking about similar targets (Mitchell et al., 2006; Krienen et al., 2010). Overall, our results suggest that regions related to mentalizing are central to the experience of empathy, potentially helping us understand the varied emotional terrain of others' everyday experiences.

When participants observed or actively empathized with a target, we also found very reliable activity in the amygdala across whole-brain contrasts, as well as in the stricter conjunction analyses. The amygdala should play a central role in empathy because it is typically active when stimuli are motivationally relevant and emotionally impactful (Ewbank et al., 2009; Adolphs, 2010; Lindquist et al., 2012). Furthermore, amygdala activation is not emotion-specific and may be part of a distributed network that helps realize core affect (Lindquist et al., 2012). Thus, our results suggest that empathy for both positive and negative emotions may heighten emotional sharing and motivational relevance, leading to increased amygdala activation.

Interestingly, only ventral AI and SA were reliably activated across emotions and attentional conditions in whole-brain analyses, suggesting that these regions may be automatically engaged during empathy. In addition, a conjunction analysis across all attentional conditions further confirmed the automatic activation of ventral AI and SA during empathy. Our results suggest that the ventral anterior insula is a core neural region for empathy across multiple emotions and is not specific to empathy for pain (Singer et al., 2009). Ventral AI may be essential to empathic processes because it is often activated by the awareness of others' affective feelings (Wager and Feldman Barrett, 2004; Craig, 2009; Lindquist et al., 2012). For both autistic individuals and controls, poorer awareness of other's emotions is related to hypoactivity in the AI (Silani et al., 2008; Uddin and Menon, 2009). Therefore, previous work that shows AI activation during empathy for pain (Singer et al., 2004) is consistent with the idea that AI may reflect a heightened awareness of others' feelings. While the septal area has not often been associated with empathy, our analyses suggest that SA should be considered an automatic and core neural region for empathy. Both prosocial behavior and maternal caregiving activate the SA (Stack et al., 2002; Gammie, 2005; Krueger et al., 2007; Inagaki and Eisenberger, 2012), suggesting that SA activation may generally signal other-oriented feelings and behaviors. In addition, different analyses on this dataset have suggested that SA activation predicts daily prosocial behavior and may signal the intention to help others (Morelli et al., in press).

When comparing passively observing and actively empathizing, whole-brain contrasts showed very few neural differences and many common neural regions across these instruction types. Common activity occurred in core empathy-related regions (i.e., MPFC, DMPFC, and TPJ), social cognition-related regions, (i.e., VMPFC/rACC, pSTS, and temporal poles) and affect-related regions (SA, ventral AI, and amygdala). Also, self-reported empathy did not differ significantly between the empathize and watch conditions. Our results preliminarily suggest that observing others engages similar empathic processes as actively empathizing with others. Because these analyses capture group-level differences, future research should explore whether neural activity during these two instruction types may differ within each individual.

We also showed that cognitive load reduces the subjective experience of empathy, as well as decreasing neural responses in several core empathy-related regions (i.e., DMPFC, MPFC, TPJ, amygdala) and social cognition-related regions (i.e., VMPFC, precuneus, posterior cingulate cortex, pSTS, and temporal poles). This finding suggests that empathy for various emotions is not entirely automatic, extending previous findings that empathy for pain and sadness are not automatic (Gu and Han, 2007; Rameson et al., 2012) and challenging the assumptions of the Perception-Action Model (Preston and De Waal, 2002). Cognitive load also increased activation in dACC and dorsal AI when compared to each of the other conditions (neutral, watch, empathize). While dACC has been reliably implicated during empathy for pain, dACC was only consistently activated during cognitive load in the current study. Thus, dACC may not be universally activated by empathic processes across emotions. It is possible that activity in dACC and dorsal AI, as well as DLPFC and VLPFC, during cognitive load reflected the increased effort and attention needed to maintain the 8-digit number in memory (Blasi et al., 2006; Woodward et al., 2006; Mulert et al., 2007). Further, cognitive load differentially activated the dorsal portion of the AI, which is associated with cognitive control processes (Wager and Feldman Barrett, 2004). In contrast, the ventral portion of the AI, typically associated with emotional awareness, was indicated in the conjunction of the watch and empathize conditions. Alternatively, dACC and AI may be performing empathic functions that are amplified under cognitive load. The role of dACC and AI during cognitive load cannot be determined from this study alone and should be explored in future research.

### Limitations and conclusion

One potential limitation of the current study design was the presentation of the watch condition in the first run, preceding the presentation of the other two conditions. Because the watch condition was meant to capture participants' completely spontaneous reactions to the emotional stimuli, we felt presenting it first was important for avoiding unwanted interference from the other instruction types. As is often the case, however, emphasizing ecological validity comes at the cost of experimental control, and this design produces an order confound. We attempted to minimize the effect of this cofound through careful pre-rating of the stimuli to insure all three conditions were otherwise as equivalent as possible. Follow-up studies in which all three conditions are intermixed will be useful in determining what, if any, effect the presentation order exerted upon the watch condition. A second limitation is that the neutral condition may not have been ideal. These photos did not directly show any faces and may not have controlled for the more detailed and varied emotional photos in the other conditions. Thus, when comparing the experimental conditions (i.e., empathize, watch, and memorize) to the neutral condition, some of the observed results—such as increased activity in the amygdala—may be due to general face processing.

In summary, the current study broadens our understanding of empathy by identifying core neural regions that underlie the empathic state. In addition, it demonstrates that empathic processes are not entirely automatic and may be disrupted by cognitive load. Lastly, the current study suggests that two key regions—the ventral AI and SA—are automatically engaged during empathy, even when attentional resources are reduced. By examining how attention impacts neural and subjective responses during empathy, we hope the current findings suggest potential ways to sustain empathy even in the face of everyday demands and distractions. Further, these findings indicate that attention impacts empathic processing and may play a role in empathic dysfunction in mental disorders such as autism.

### Conflict of interest statement

The authors declare that the research was conducted in the absence of any commercial or financial relationships that could be construed as a potential conflict of interest.
